# Broadband optical spin dependent reflection in self-assembled GaAs-based nanowires asymmetrically hybridized with Au

**DOI:** 10.1038/s41598-021-83899-2

**Published:** 2021-02-22

**Authors:** Emilija Petronijevic, Alessandro Belardini, Grigore Leahu, Teemu Hakkarainen, Marcelo Rizzo Piton, Eero Koivusalo, Concita Sibilia

**Affiliations:** 1 Dipartimento di Scienze di Base ed Applicate per l’Ingegneria, Sapienza Università di Roma, Via A. Scarpa 16, 00161 Rome, Italy; 2 Optoelectronics Research Centre, Physics Unit, Tampere University, Korkeakoulunkatu 3, 33720 Tampere, Finland

**Keywords:** Nanoscience and technology, Optics and photonics

## Abstract

Hybridization of semiconductor nanostructures with asymmetric metallic layers offers new paths to circular polarization control and chiral properties. Here we study, both experimentally and numerically, chiral properties of GaAs-based nanowires (NWs) which have two out of six sidewalls covered by Au. Sparse ensembles of vertical, free-standing NWs were fabricated by means of lithography-free self-assembled technique on Si substrates and subsequently covered by Au using tilted evaporation. We report on optical spin-dependent specular reflection in the 680–1000 nm spectral range when the orientation of the golden layers follows the rule of extrinsic chirality. The analysis shows reflection peaks of the chiral medium whose intensity is dependent on the light handedness. We further propose a novel, time-efficient numerical method that enables a better insight into the far-field intensity and distribution of the scattered light from a sparse NW ensembles. The measurements done on three different samples in various orientations show good agreement with theoretical predictions over a broad wavelength range.

## Introduction

Semiconductor nanowires (NWs) have been extensively investigated as good candidates for building blocks in nanophotonics and nanoelectronics because of their unique optoelectronic properties. III–V semiconductor NWs, made of high quality, high refractive index materials with controllable dimensions at the nanoscale, can confine and manipulate the electromagnetic fields of the visible and near infrared wavelengths. Specifically, coupling of the light to the resonant modes of the nanostructure^1,2^ can tailor the light absorption^3–5^, reflection^6^, emission^7–9^ and lasing^10,11^, important for both photodetector and source applications. Furthermore, evanescent modal fields in semiconductor NWs have been shown to carry transverse spin angular momentum (SAM)^12,13^, therefore it came naturally to extend NW investigation to spin-related phenomena, chirality and circular dichroism (CD) i.e. different interaction with circular polarizations (CP) of opposite handedness. Namely, regions of enhanced near-field chirality were numerically shown in periodic arrays of Si NWs^14^ and Si NW dimers^15^, as well as in individual hybrid Au-GaAs NWs^16^. In order to assess chiral behaviour in the far-field, the shape of the nanostructure can be made chiral, in which case they exhibit intrinsic chirality^17,18^. Intrinsic chirality in NW-based samples was demonstrated in the far-field in Ref.^19^: the authors fabricated left and right spiral metal-GaN NW lasers by lithography, and demonstrated high CD in ultraviolet lasing at room temperature.

On the other side, CD can be induced even in achiral nanomaterials fabricated by simpler, self-assembled techniques, given that they possess symmetry breaking in the interaction with CP. This so called *extrinsic chirality* is present when the light wave-vector, the surface normal of the sample, and the average orientation of the sample asymmetry, form a non-planar triad of vectors^20,21^. Experimentally, extrinsic chirality is found in samples covered by some asymmetric layer, properly oriented and brought to interaction with CP at oblique incidence. We recently showed that vertical ensembles of GaAs-based NWs, fabricated by self-assembled, lithography-free growth on Si substrates, can be asymmetrically covered by a thin layer of Au (three out of six sidewalls) and properly oriented to differently absorb left and right CP (LCP and RCP, respectively). CD was measured at single wavelengths in absorption by means of photo-acoustic technique^22^, and further demonstrated in photoluminescence emission^23^, and in second harmonic generation^24^.

In this work, we experimentally perform a broadband characterization of chirality in reflection in GaAs-based NW ensembles, where a thin Au layer covers two out of six NW sidewalls. We investigate three samples and show that when the extrinsic chirality condition is fulfilled, the light intensity reflected to the far-field becomes spin-dependent in a broader spectral range, exhibiting interference minima and maxima from an effective chiral medium. We further propose a time-efficient numerical method for investigation of near- and far-field properties in non-periodic, self-assembled ensembles of sparse NWs; we show that this way of modelling can be used to study and optimize influence of various fabrication parameters on the reflection and the chiral response in a desired broad wavelength range.

## Experimental results

**Figure 1:**
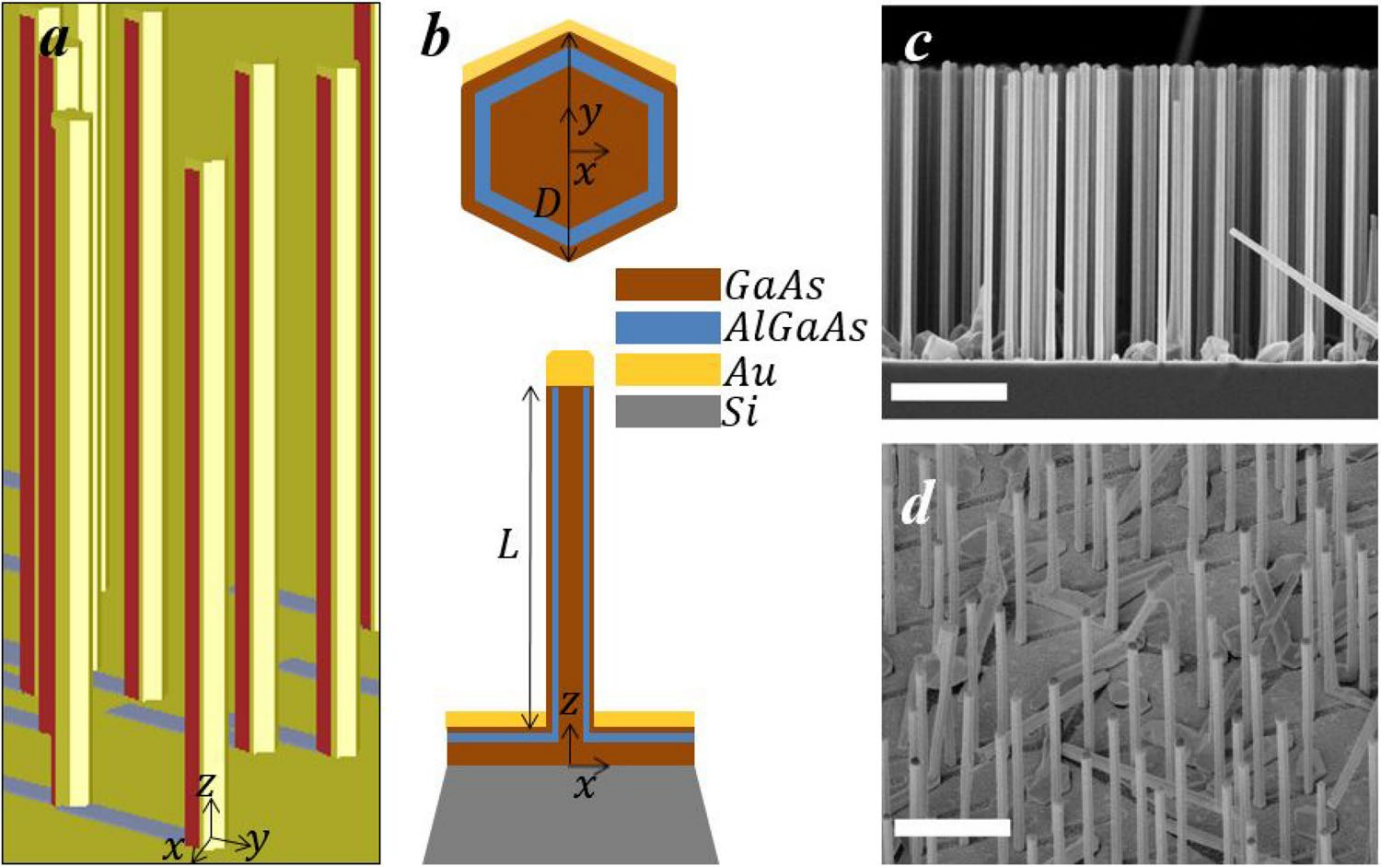
(**a**) 3D schematic of the NW ensemble. (**b**) Cross section of one NW in the xy and xz planes, with overall diameter D, and NW length L. (**c**) Side-view SEM image of Sample B0. (**d**) Tilted SEM image of 3D distribution of Sample B1. Scale bar equals 2 μm.

We investigate three samples, A1, B1, and B0, with geometric parameters given in Table 1. The samples were grown by molecular beam epitaxy on p-Si(111) wafers, using lithography-free Si/SiOx patterns to define the nucleation sites^25^ (see “Methods”). This technique results in ensembles of vertical semiconductor NWs which have tailorable density and highly uniform dimensions^26,27^. The NWs are made of GaAs core, AlGaAs shell and GaAs supershell. For the formation of an asymmetric Au layer in Samples A1 and B1, there is a second step which involves the Au evaporation on the tilted NW ensemble. This step results in NWs which have two out of six sidewalls covered by a thin (~ 17 nm) Au layer, ~ 80 nm thin Au cap on the top, and ~ 80 nm Au layer formed on the substrate and interrupted by the NW shadow (Samples A1 and B1). A 3D sketch on such ensemble is shown in Fig. 1a, while Fig. 1b shows xy and xz cross-sections of one NW. Sample B0 has the same parameters as Sample B1, except that it was not covered by Au. A side-view SEM image of Sample B0, and a tilted-view SEM image of Sample B1 are shown Fig. 1c,d, respectively.

**Table 1:** Characteristic geometric parameters for the three samples.

Sample	L (nm)	D (nm)	ρ_s_ (cm^−2^)	
A1	4400	140	1 × 10^8^	With Au
B1	5150	150	8 × 10^7^	With Au
B0	5150	150	8 × 10^7^	No Au

**Figure 2:**
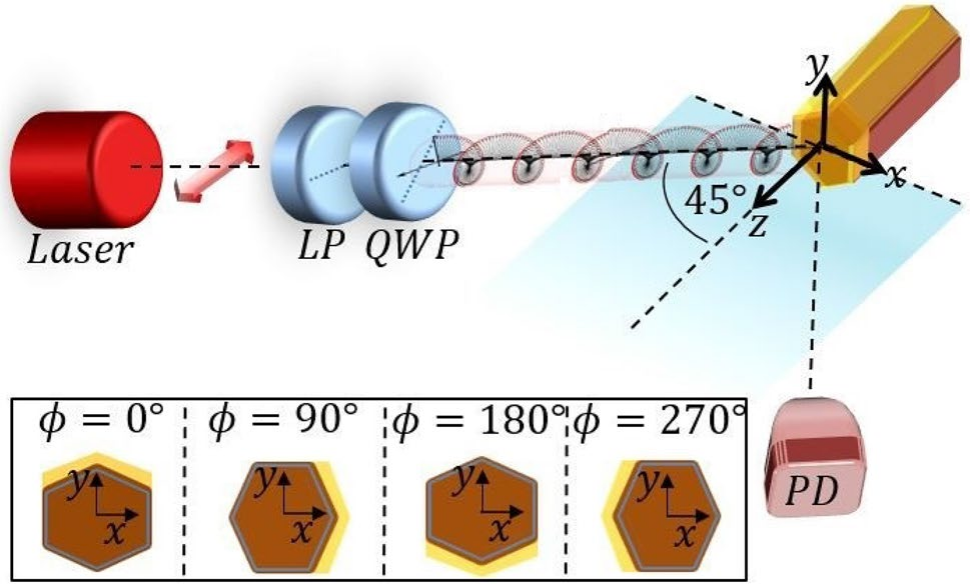
Schematic of the experimental set-up for extrinsic chirality in reflection. Inset: orientations of the NW ensemble, given as a clockwise rotation around the z-axis. The incidence angle is fixed at 45°.

The samples are excited by a near-infrared laser, widely tuneable in the 680–1000 nm range (see “Methods”). The laser light enters a linear polarizer (LP) and a rotating quarter wave plate (QWP), which defines the spin of the impinging light. The QWP angle of 45° (− 45°) carries the SAM of –ħ (ħ), and defines RCP (LCP). All the measurements are carried out at room temperature using 45° angle of incidence. In Fig. 2 a simplified schematic of the experimental set-up is shown. The xz plane of incidence contains the light wave-vector and the surface normal of the sample, which is fixed in the z-direction. In order to induce the extrinsic chirality, the average normal of the asymmetric layer must lie in a different plane. In the inset of Fig. 2 we show the possible rotations of the sample, and assume that the extrinsic chirality is possible when the average Au layer normal points in the y-direction (ϕ = 0° or ϕ = 180°). The reflected signals are measured by a specularly positioned Si photodiode (PD). All the reflection measurements are normalized with respect to the reflection of an Au mirror.

**Figure 3:**
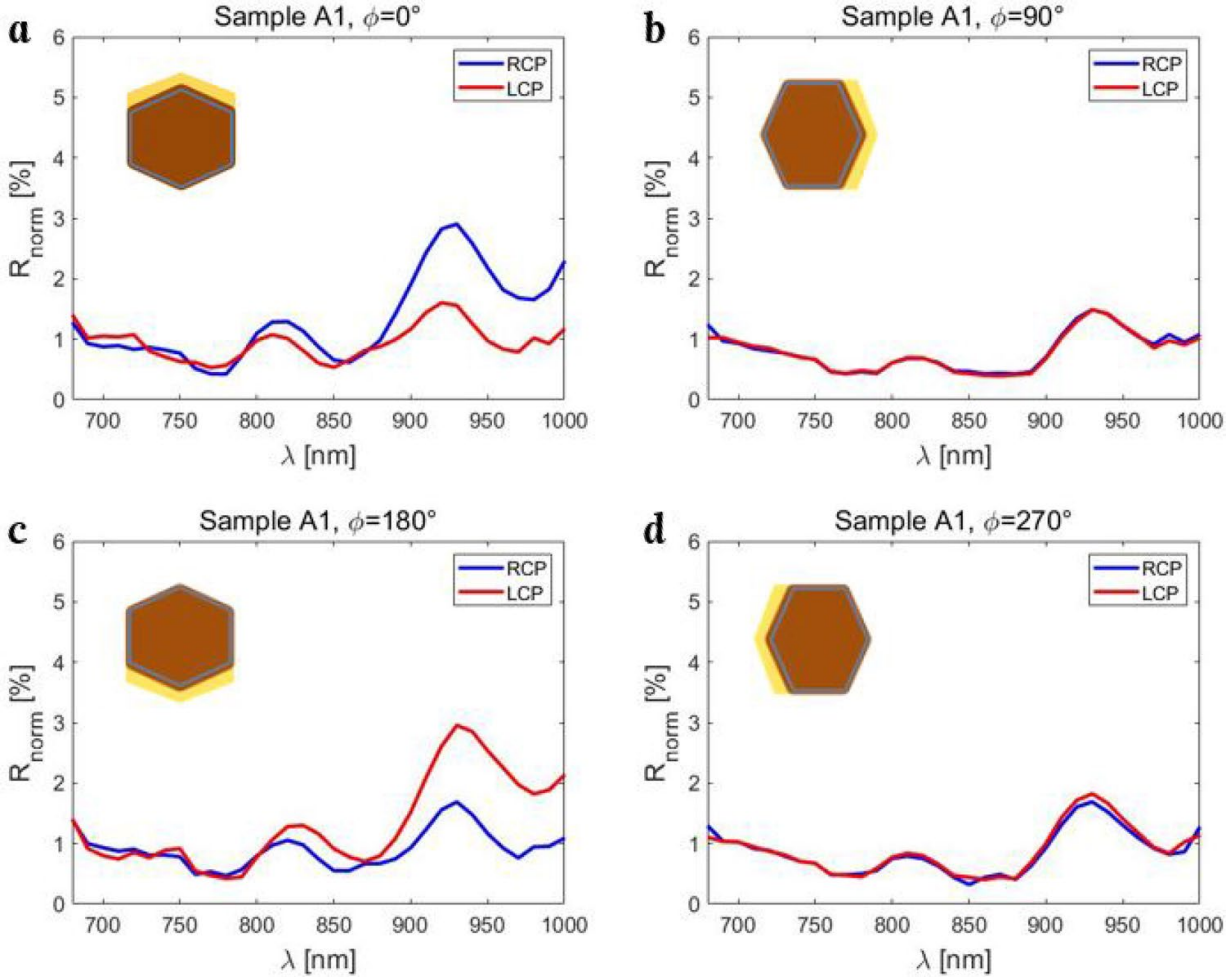
RCP and LCP reflection spectra for various orientations of Sample A1: (**a**) ϕ = 0°; (**b**) ϕ = 90°; (**c**) ϕ = 180°; (**d**) ϕ = 270°.

In Fig. 3a we report on reflection measurements with LCP and RCP excitations, for Sample A1 orientation of ϕ = 0°. Both excitations result in characteristic reflection peaks and dips, which are spectrally mostly at equal positions. However, as the average Au surface normal points in the positive y-direction, the non-planar triad of vectors is formed, and RCP leads to higher reflection than LCP. Moreover, the difference is larger for longer wavelengths. For the ϕ = 90° orientation, the average surface normal lies in the incidence plane, therefore LCP and RCP are equally reflected, Fig. 3b. Inverting the Au direction from ϕ = 0° to ϕ = 180° in Fig. 3c leads to the opposite behavior, where RCP becomes less reflected, and LCP (RCP) spectral shape becomes almost equal to RCP (LCP) from Fig. 3a. This is a direct consequence of the non-planar triad inversion in extrinsic chirality, and we previously noticed it in photoacoustic absorption measurements^22^. Finally, extrinsic chirality vanishes again for ϕ = 270°, Fig. 3d.

**Figure 4:**
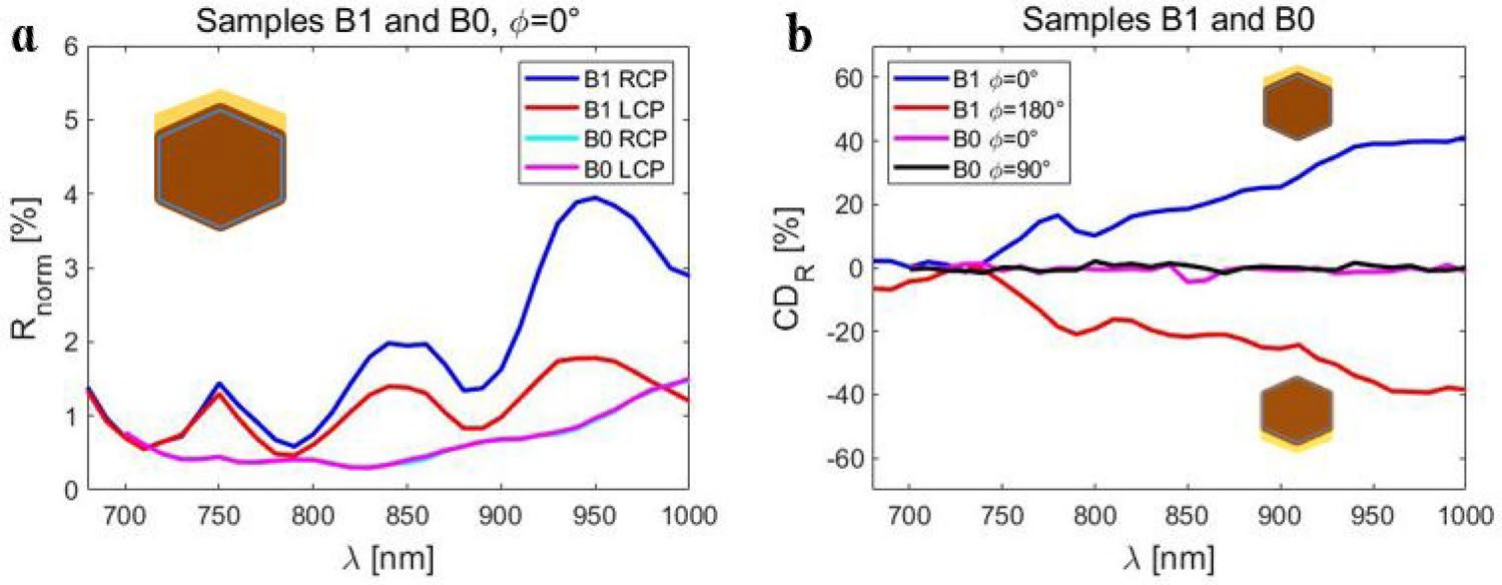
(**a**) Samples B1 and B0: RCP and LCP reflection spectra for ϕ = 0°. (**b**) CD_R_ for various orientations of Samples B1 and B0. The inversion of the CD_R_ sign between ϕ = 0° and ϕ = 180° is characteristic proof of extrinsic chirality.

Next, we perform the same measurements on Sample B1, Supplementary Fig. S2. The reflection peaks are higher than in Sample A1, and spectrally red-shifted. The extrinsic chiral behavior holds true. In Fig. 4a we compare Samples B1 and B0 at ϕ = 0°. Without the Au layer, both LCP and RCP excitation lead to equal reflection in the broad spectral range in Sample B0. The overall reflection of such sparse ensemble without Au is lower, and there are no pronounced reflection peaks and dips as in Samples A1 and B1. We further define a figure of merit for CD in reflection, CD_R_:

1 $$ CD_{R} = 100\frac{{R_{RCP} - R_{LCP} }}{{R_{RCP} + R_{LCP} }}. $$

In Fig. 4b, we clearly see the almost perfect inversion of CD_R_ between ϕ = 0° and ϕ = 180° in Sample B1. CD_R_ reaches ± 40% in the highly reflective spectral range, for wavelengths above 930 nm. As expected, CD_R_ equals 0 in the whole spectral range for Sample B0, for both ϕ = 0° and ϕ = 90°. Sample A1 generally exhibits slightly lower CD_R_ in the broadband range, but with pronounced oscillatory behavior, Supplementary Fig. S3. We can conclude that the extrinsic chirality in these samples are solely due to the two Au sidewalls.

Even though there are high scattering losses decreasing the specular reflection efficiency, the extrinsic chirality is demonstrated in the broadband range, and of CD_R_ magnitudes comparable to our previous works^21–23^. In the following, we propose a numerical method that enables us to gain insight into a complex electromagnetic behavior of random chiral NW ensembles, and visualize how the spin of the incident light controls the light-NW ensemble interaction in the near- and far-field.

### Numerical investigation

Strong reflection oscillations and strong birefringence in non-periodic ensembles of semiconductor core–shell (GaP-SiO_2_) NWs were previously measured in Ref.^28^, and explained by means of the Maxwell–Garnett effective medium theory for core–shell cylinders. The work proved that the oscillations, similar to the ones presented in Figs. 3 and 4a, arise because the NW ensemble acts as a Fabry–Pérot cavity between its interfaces with the substrate and the air. These oscillations strongly depend on the NW height, diameter, density and surrounding medium, which renders such nanomaterials promising for sensing applications. More recently, authors in Refs.^29,30^ investigated similar reflectance modulation in dense ensembles of self-assembled InAs NWs, and proposed them for sensing applications; since the effective medium theory fails to account for the scattering within the NW ensemble at shorter wavelengths, the authors resorted to numerical simulations to completely recover the reflectance spectral features. Even though in this work we investigate hybrid metal–semiconductor samples of much lower density, in general the reflection behavior for Samples A1 and B1 is in agreement with the previously published ones; we believe this is due to the presence of the plasmonic layer which enhances the effective refractive index of the medium. Clear reflection oscillations are present in the near-IR range for both LCP and RCP, while the intensity of the peaks decrease with shorter wavelengths due to the semiconductor absorption and scattering effects. It is worth noting that such resonant behavior is enabled by the size uniformity of the individual NWs in our samples, while being robust with respect to the aperiodic NW arrangement; namely, our fabrication technique has been shown to provide sub-Poissonian length distribution^26,27^, thus enabling stable parameters of the effective Fabry–Pérot cavity between the air and the substrate.

**Figure 5:**
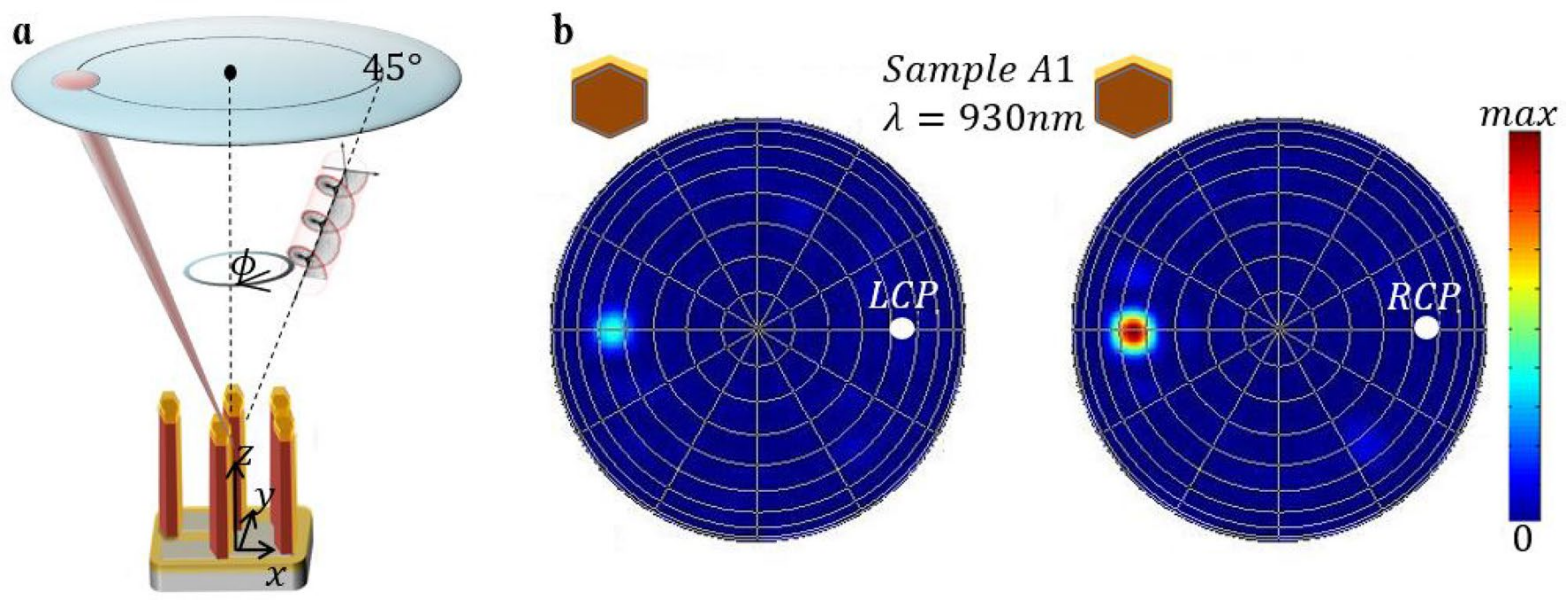
(**a**) Sketch of the simulation set-up. (**b**) Far-field for LCP and RCP excitations of Sample A1, at ϕ = 0° and 930 nm. White circles denote the position of the incoming field.

In order to correctly account for the presence of asymmetric Au shell and visualize the near-field and the far-field dependence on the excitation spin, we propose a quasi-random numerical approach for the NW ensemble simulation in Lumerical^31^. The commercial 3D Finite Difference Time Domain (FDTD) solver allows for the excitation and full-wave propagation of the electromagnetic wave across such complex medium. In our previous work, the photo-acoustic absorption spectra were easily reproducible with single NW simulations, while the scattering and the NW neighbor interactions were negligible^5,22^. Here, instead, one is to model the light intensity which is specularly scattered by the NW ensemble, and its dependence on the excitation spin, as shown in Fig. 5a. The NW ensemble is excited by a broadband CP wave, the scattered light is projected to the far-field, and the simulation result is the light intensity, reflected to the specular cone and integrated over its surface in the far-field. Single NW simulations in this case are not able to reproduce the experimental data, while simulations of many NWs in low density ensembles require high memory and time cost in broadband calculations (see “Methods” and Supplementary Information). We therefore performed convergence tests by increasing the number of NWs (hence the FDTD volume), and obtained convergence starting from 25 NWs. Therefore, the FDTD region contains 5 × 5 vertically standing NWs, randomly positioned in 25 subcells, so that we take into account the known densities of the three samples (Supplementary Fig. S4). In Fig. 5b we show the far-field result for two simulations (LCP and RCP excitation) of Sample A1, oriented at ϕ = 0°; the results are extracted at wavelength of 930 nm, which corresponds to CD_R_ maximum (Supplementary Fig. S3). Clearly, the incident light spin controls the intensity of the reflected light.

**Figure 6:**
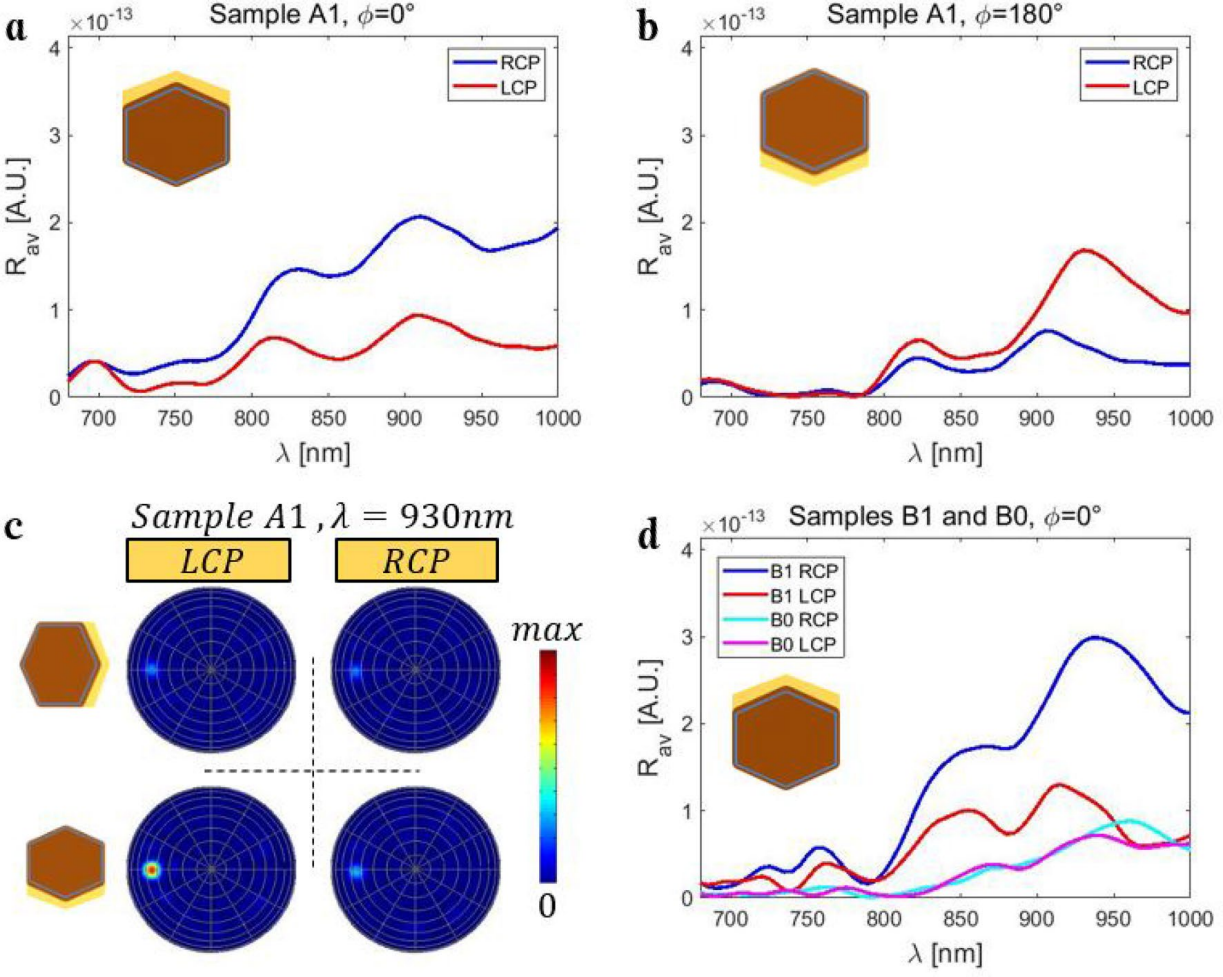
RCP and LCP far-field reflection spectra for orientations of Sample A1: (**a**) ϕ = 0°, and (**b**) ϕ = 90°. (**c**) Far-field for LCP and RCP excitations at 930 nm, for Sample A1 at ϕ = 90° (top) and ϕ = 180° (bottom). (**d**) RCP and LCP far-field reflection spectra for Samples B1 and B0, at ϕ = 0°.

Next, we for each wavelength in the broadband simulation, we project the back-scattered light to the far-field and integrate the intensity across the 1° cone. The results for Sample A1 and ϕ = 0°, shown in Fig. 6a, resemble those in Fig. 3a. We then rotate each of 25 NWs for 180° around z-axis, and see that RCP and LCP behavior inverts as expected, Fig. 6b. Therefore, by playing with the incident polarization and the NW orientation, one can control the light intensity in the far-field. In Fig. 6c we show the far-field maps for Sample A1, and orientations ϕ = 90° and ϕ = 180°, at 930 nm (the color scale is equal to the one in Fig. 5b). For ϕ = 90°, the reflection is much lower (Fig. 3b) and equal for LCP and RCP. However, for ϕ = 180°, LCP is more reflected than RCP. Finally, we perform simulations of Samples B1 and B0, at ϕ = 0°. Again, for Sample B1 the agreement with the experimental data is good, regarding both spectral positions and intensity (the intensity scale is the same as Fig. 6a,b).

For Sample B1 reflection peaks are stronger and red-shifted with respect to Sample A1; the origin of this change is the interplay between the change of the diameter, density or the NW layer thickness. Even though the NWs in Sample B1 have thicker diameter, their density is lower; the corresponding volume filling factors for Sample A1 and B1 are 1.96% and 1.8%, respectively. We believe that this is the reason for the higher reflection (Supplementary Fig. S5). On the other hand, the longer NWs in Sample B1 increase the optical thickness of the NW layer, and are responsible for the red-shifted reflection.

Since the origin of the chiral effects lies in the symmetry breaking by the Au sidewalls, we further numerically investigate how the deposited Au thickness and shape of the sidewalls influence the results. First, we tackle the problem of the shadow effect due to the tilted deposition. The shadow from the NW interrupts the Au layer on the substrate (grey rectangles in Fig. 5a), and it has been already taken into account in simulations (Methods). However, the two NWs in a random ensemble can be grown close enough, so that the shadowing effect of one effectively decreases the Au sidewall length of the other one. However, we believe that such NW neighbors do not have a significant effect on the results, as explained in the Supporting Information, and shown in Supplementary Fig. S6. We next investigate the Au thickness influence. Again, the overall chiral behavior and position of the reflection peaks do not significantly differ for Au layers thicker than 15 nm, Supplementary Fig. S7, while in the longest wavelength part of the investigated range, thicker sidewalls provide higher reflection of RCP. In Supplementary Fig. S8 we include the response of a NW ensemble where NWs have the same parameters of Sample B1, but they have three out of six Au-hybridized sidewalls. The average surface normal now points in the direction of the middle Au sidewall; as expected, when this is in the y-direction, the extrinsic chiral behavior holds, leading to somewhat lower reflection. These parameters can be further used for CD_R_ optimization (Supplementary Information). Moreover, this study confirms the robustness of our design and self-assembled fabrication technique.

**Figure 7:**
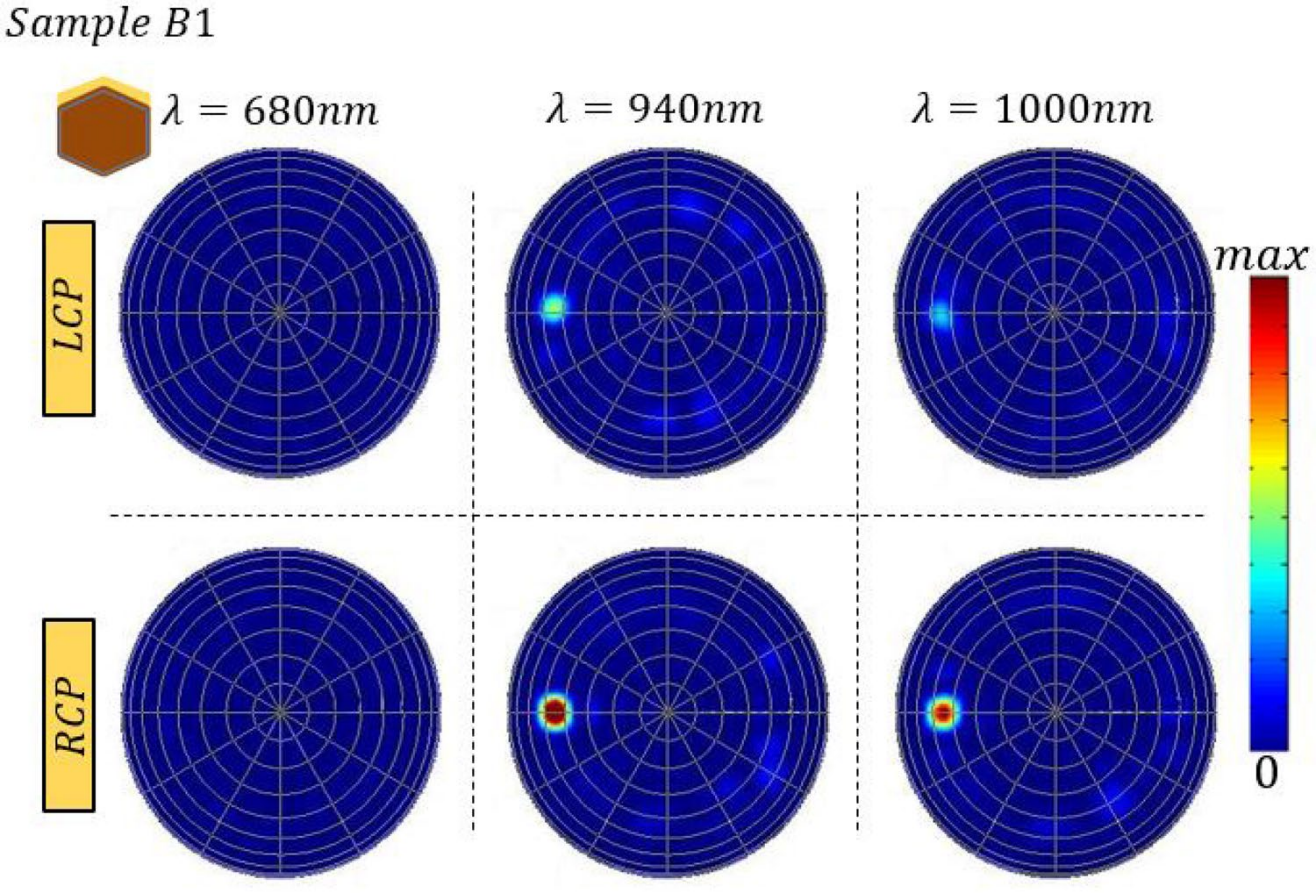
Far-field maps of Sample B1, at ϕ = 0°, for LCP and RCP excitation at wavelengths of 680 nm, 940 nm and 1000 nm.

Further, we show how the excitation wavelength controls the far-field intensity for Sample B1, by far-field visualizations at specific wavelengths, Fig. 7. At 680 nm, there is a high absorption in the sample, so the reflected part of the light is low and similar for LCP and RCP. At 940 nm, the far-field difference reaches its maximum in both experiment (Fig. 4a) and simulations (Fig. 6d). The near-field confinement at this wavelength is investigated in Supplementary Information; while LCP excitation leads to complex pattern of the electromagnetic field partially transmitted through the NW layer and absorbed by the Si substrate, for the RCP excitation there is a constructive interference with the chiral medium, leading to the reflection maximum (Supplementary Fig. S9–10). Finally, at 1000 nm, the NW ensemble is in the range of reflection with characteristic maxima and minima, and both LCP and RCP have lower intensity than at 940 nm.

Since single NW dimension forming the chiral medium strongly affect the reflection intensity and CD_R_, in the Supplementary Information we further investigate several different geometries for a possible optimization of CD_R_. In Supplementary Fig. S11 we show how the extrinsic chirality can be tuned by changing the NW length and diameter according to fabrication dependencies characterized in Ref.^27^. We also report on the absorption CD, which we defined in our previous works^22^ and measured by photo-acoustic technique. This parameter can be further improved by optimizing the angle of incidence, Supplementary Fig. S12. Finally, we believe that both CD_R_ and absorption CD can be highly influenced by the presence of chiral medium around the NWs. On the other side, a reflection-based platform can be of interest for chiral detection applications where a simple in-plane rotation of aperiodic NW ensembles with asymmetric plasmonic shells changes CD_R_ sign, thus enabling differential measurements.

## Discussion

Our results have demonstrated that the extrinsic chirality can be obtained in the far-field from aperiodic sparse ensembles of GaAs-based NWs asymmetrically covered by Au. We have measured the specular reflection, and showed that it is dependent on the spin of the excitation in a broad near-IR range. Moreover, we have proposed time-efficient simulation method which reveals near- and far-field response of many NWs. Since the simulations are in great agreement with the experiments, they can be used to further optimize the NW dimensions for the strong CD in the spectral range of interest. Finally, we strongly believe that self-assembled NW samples, asymmetrically covered by Au, can be used for chiral light manipulation in both near and far field.

## Methods

### NW fabrication

The investigated samples consist of vertically standing NWs grown by molecular beam epitaxy on Si(111) wafers using lithography-free Si/SiOx patterning technique for defining the nucleation sites^25^. The GaAs core, having a diameter of 130 nm, was first grown by self-catalyzed vapor–liquid-solid growth. Then the Ga catalyst droplet was consumed in As2-flux in order to terminate axial growth. Subsequently, the Al_0.3_Ga_0.7_As shell and GaAs supershell were grown by vapor–solid method. The detailed growth procedure for the core–shell-supershell nanowire structures is presented in Ref.^25^. The nominal thicknesses of the shell layers were varied between Samples A1 and B1 in such way that their overall diameters are 140 nm and 150 nm, respectively. As a result of axial elongation during shell growth, the average length of the NWs in Sample A1 is 4400 nm, while in Sample B1 it is 5150 nm. The diameter and length dimensions are average values obtained from investigation of a large number of NWs from side-view scanning electron microscope images (see Fig. 1c).

The semiconductor–metal hybrid structures were obtained by growing a thin Au layer on the NWs using electron beam evaporation. The NW sample was tilted in a 14° angle with respect to the nanowire axis in order to obtain the asymmetric structure presented in Fig. 1a,b. The tilt angle was chosen based on the average length and nearest neighbour statistics of the NWs (see Supplementary Fig. S1 in the Supplementary Information for more details), thus minimizing the shadowing effects. The azimuthal direction of the Au flux and the deposition time were chosen in such way two of the six (110) type NW side facets would get nominally a 17 nm thick layer while the other four (110) facets remain Au-free.

### Reflection measurements

The samples were excited by a widely tuneable near-infrared laser (Chameleon Ultra II by Coherent Inc., Santa Clara, CA, USA), which has the pulse duration of 140 fs, and the repetition rate of 80 MHz. In order to perform linear characterization of reflection, a mechanical chopper at 70 Hz was put on the laser’s exit. The output laser power was decreased to 4% by a beam-splitter, and further by a neutral density filter of 0.62.

### Numerical calculations

We have used Lumerical Finite Difference Time Domain (FDTD) solver to simulate the far-field reflection distribution spectra of ensembles of 25 NWs. Each NW has GaAs core, AlGaAs shell, and GaAs supershell, and the ensemble stands on a semi-infinite Si substrate. These NWs are positioned over 5 × 5 subcells, where each NW takes random position in the xy plane over its cell. For Sample A1, one contains 1 NW/μm^2^, while the lower density of Samples B1 and B0 require larger subcell (1 NW/(1.25 μm^2^)). Complex refractive index of GaAs was taken from Ref.^32^, while those of Au and Si were taken from Lumerical material database, and fitted over the 680–1000 nm range. We include the shadowing effect due to the Au evaporation, by etching the Au layer on the substrate with a rectangle of sides D and L·tan(14°). In order to simulate CP excitation from the experiment, the NW ensemble was excited by two BFAST sources; the sources were perpendicular to each other, with a phase difference of 90° (− 90°) in order to simulate RCP (LCP), while the other properties of the sources were equal. The far-field was projected from the near-field monitor placed above the source.

## Supplementary Information

Supplementary Information.
