# Supplementary material for: Broadband optical spin dependent reflection in self-assembled GaAs-based nanowires asymmetrically hybridized with Au
Source: Sci Rep. 2021 Feb 22;11:4316. doi: 10.1038/s41598-021-83899-2 (PMC7900205; doi:10.1038/s41598-021-83899-2)
Supplement: Supplementary file 1 — Supplementary Information. [file 41598_2021_83899_MOESM1_ESM.docx]

**Broadband optical spin-dependent reflection in self-assembled GaAs-based nanowires asymmetrically hybridized with Au**

**– SUPPORTING INFORMATION**

**Emilija Petronijevic^a,*^, Alessandro Belardini^a^, Grigore Leahu^a^, Teemu Hakkarainen^a,b^, Marcelo Rizzo Piton^b^, Eero Koivusalo^b^, Concita Sibilia^a^**

a. Dipartimento di Scienze di Base ed Applicate per l’Ingegneria, Sapienza Università di Roma, Via A. Scarpa 16, 00161 Roma, Italy

b. Optoelectronics Research Centre, Physics Unit, Tampere University, Korkeakoulunkatu 3, 33720 Tampere, Finland

1. **Neighbor distance statistics and Au flux shadowing**

Figure S1 presents a statistical analysis of Au flux shadowing for sample B1, with mean NW length L=5150 nm. In case of Au flux angle of 14°, as used in the experiments, the length of the shadow cast by a single NW in the horizontal plane is 1280 nm. From Fig. S3(c) we can see that there are on average three NWs within this shadowing distance. However, we should note that those three neighbors can cause shadowing only if they are in the direction from which the flux comes, as illustrated in Fig. S1(d). Consequently, there is a finite range of azimuthal angles that satisfy this condition for given NW diameter and distance. By accounting for this criterion, we estimate that shadowing probability for NW distance *x*=747 nm (corresponding to the mean value of nearest neighbor distance) is 6% assuming NW diameter D=150nm. From this statistical analysis we can thus conclude that, although the nearest neighbor distance is shorter than the shadow length, majority of the NWs in the ensemble experience no Au-flux shadowing by the other NWs.

**
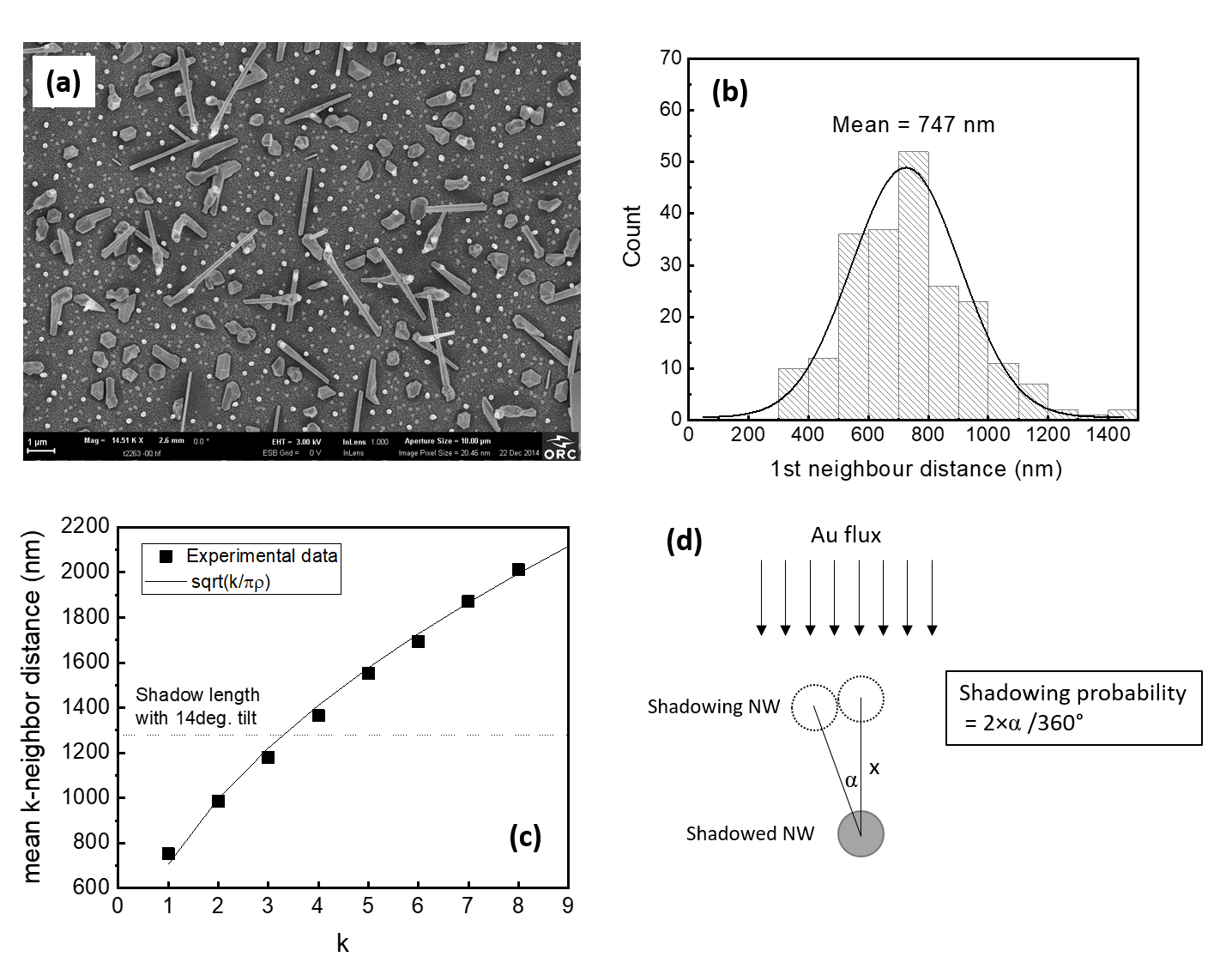
**

Figure S1: Statistical analysis of Au-flux shadowing by the neighboring NWs. (a) The vertical NWs are seen as bright spots in the top view SEM picture of Sample B0; (b) The nearest neighbor distribution (k=1) obtained from the SEM data; (c) The mean distance of the k-th neighbor obtained from the SEM data. The solid line represents the theoretical prediction based on the assumption that a circle with a radius equaling to the k-th neighbor distance encloses k neighboring nanowires. Therefore, the distance of the k-th neighbor is expected to be $\sqrt{k/{\pi\rho}}$, where *ρ* is the area density of the NWs. The horizontal solid line in (c) is the length of the shadow in sample B1, calculated for NW length L=5150 nm Au flux angle of 14°. (d) A top-view sketch illustrating the range of azimuthal angles α that can cause Au flux shadowing when the flux direction remains constant.

1. **Experimental data**


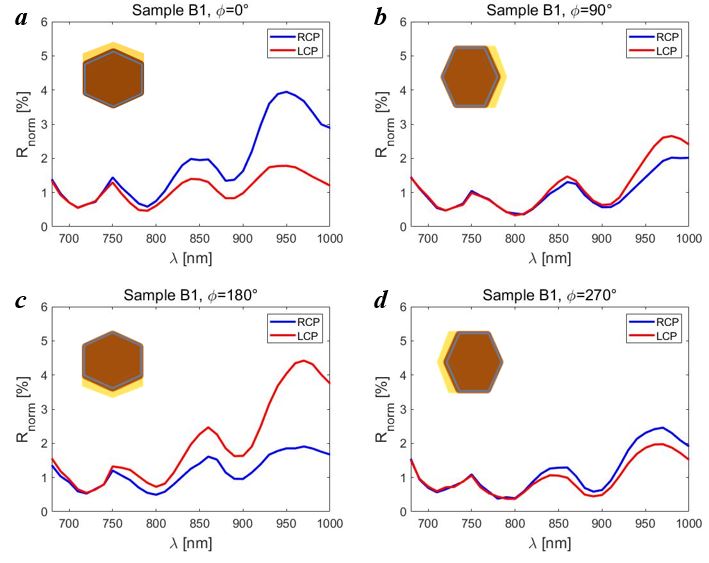


Figure S2: RCP and LCP reflection spectra for various orientations of Sample B1: (a) ϕ=0°; (b) ϕ=90°; (c) ϕ=180°; (d) ϕ=270°.


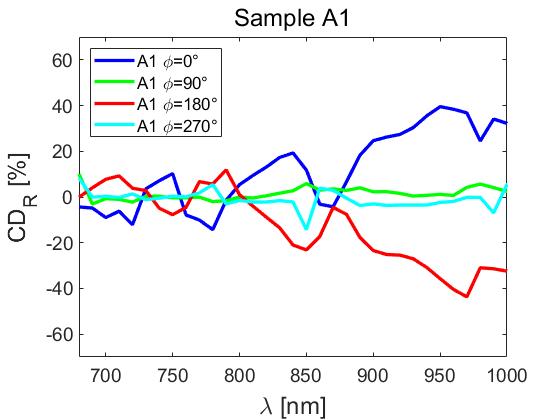


Figure S3: CD_R_ for various orientations of Sample A1. The inversion of the CD_R_ sign between ϕ=0° and ϕ=180° is characteristic proof of extrinsic chirality.

1. **Numerical calculations in Lumerical, Inc**

The 3D Finite Difference Time Domain (FDTD) method in Lumerical [1] is a powerful numerical tool which solves Maxwell’s equations in complex nanoscaled materials. We previously used it to model absorption behavior in GaAs-based NWs at normal incidence [2], as well as chiral behavior in similar samples covered by Au [3]. In those works, a single NW or a NW pair (positioned according to nearest neighbor distribution) was excited by a total-field-scattered-field (TFSF) source, and the absorption cross-section was collected in the total-field region inside the source. In this work, we investigate broadband near-IR coupling of the LCP/RCP with the NW ensembles at oblique incidence. The goal is to visualize the near-field phenomena and their implications on the far-field distribution of the reflection, and to reproduce the experimental data. Previous modelling in this case raises many problems. First, due to the complexity of the hybridization with Au, the far-field scattering due to the LCP/RCP excitation of sparse NW ensembles at oblique incidence cannot be explained by a single NW response; therefore, the FDTD region must contain many NWs. Secondly, while at normal incidence TFSF source in Lumerical can be used as a broadband source, at oblique incidence there is a common error: since the in-plane vector must remain equal at all wavelengths, the actual injection angle becomes a function of the wavelength (for this reason we used oblique TFSF source at single wavelengths in [3]). Finally, electromagnetic (EM) fields collected in the near-field monitor standing above the source (in the reflection region of FDTD) are to be projected to the far-field; for converging and stable results, this step requires EM fields tending to zero value on the borders of the monitor. This further leads to FDTD xy-surface being much wider than the one of the actual NW ensemble, making the computational volume extremely large. Therefore, by using TFSF source, we would need to run highly memory- and time-consuming simulations at each wavelength for LCP and RCP excitation.

Instead, we resort to a different approach which makes use of Lumerical’s Broadband Fixed Angle Source Technique (BFAST). BFAST sources ensure that in a single simulation run, the whole 680-1000 nm spectrum gets injected at 45°. As with plane-wave sources, two perpendicular BFAST sources with phase offset of ±90° are combined to model CP wave. CP wave then excites the NW ensemble from the air, as in the experiment, while the reflection monitor above the BFAST sources collects the near-field data and projects it to the far-field (Fig. 5a). Further, in the xy-plane, the FDTD domain is defined by the number of NWs needed for the convergence and for the modelling of the whole ensemble. We assume that in sparse NW ensembles reflection features depend on the NW density and dimensions rather than on the actual nearest neighbor distribution. Therefore, we take surface containing N^2^ NWs, divide it into N^2^ squares, and randomly position one NW inside the square. The schematic of this procedure is shown in Fig. S4; in the sketch we used a top-view SEM image of the sample with the same density of Sample A1, previously published in [2]. For Sample A1, the surface density is 1 NW/µm^2^, therefore the square has surface of 1 µm^2^. For Samples B1 and B0, the surface density is 0.8 NW/µm^2^, therefore the square has surface of 1.25 µm^2^. In the post-processing code, we extract the EM power scattered to the specular cone (Fig. 5a) at each wavelength, as explained in the main manuscript. We then perform convergence testing and find that for N=5, a single broadband simulation already reproduces the experimental data; moreover, it lasts up to 3h, which makes it several times faster than the single wavelength TFSF simulation with the same number of NWs. Finally, such simulations allow for the investigation of NW geometry influence on the near- and far-field spin-dependent properties.


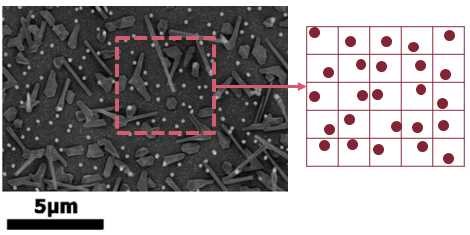


Figure S4: Schematic of the procedure for the modelling of NW ensemble. SEM image was taken from the Supporting Material of our previous work [2].

We first investigate influence of the main geometric parameters on the extracted far-field spectra for RCP and LCP excitations. We fix the density of the NWs to be 1 NW/µm^2^, oriented at ϕ=0°. In Fig. S5a-b we plot the dependence on the NW length, by fixing other parameters to the ones of Sample A1. We clearly see the red shift of the interference pattern due to the increase of the NW length, while the overall intensity does not change considerably. Next, we fix the NW length to 4400 nm, and plot the dependence on the NW diameter, Fig. S5c-d. Here, instead, there is an evident change in the intensity due to the fact that larger diameter effectively increases filling factor of the NW material in the FDTD region: NWs with larger diameters absorb more light, hence leading to lower reflection.


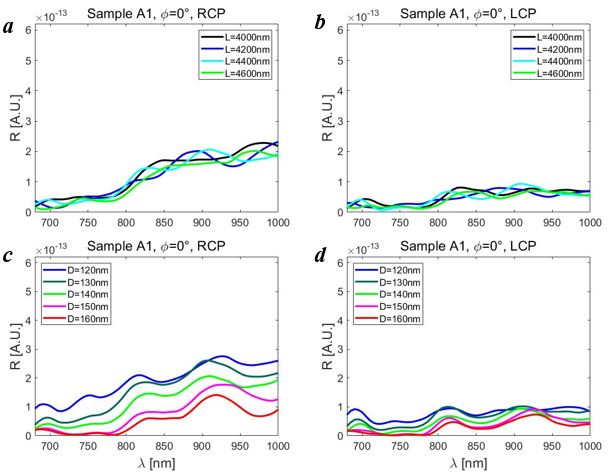


Figure S5: Far-field reflection spectra at ϕ=0°, as a dependence on: (a) length L at RCP excitation; (b) length L at LCP excitation; (c) diameter D at RCP excitation; diameter D at LCP excitation. Constant parameters are fixed to the ones of Sample A1.

Since the origin of the chirality lies in the thickness and geometry of the Au sidewalls, we next investigate these parameters in more detail. First, even in low density self-assembled NW ensembles and at low Au deposition tilt angles, several micron long NWs can be positioned close enough so that the shadowing effect of one NW influences the Au sidewall length of the other NW. We numerically investigate this effect by defining the Au-free part of the NW (offset from the bottom) as t_off_, as indicated in Fig. S6a. We keep the parameters of Sample B1 oriented at ϕ=0°, and change t_off_. In Fig. S6b-c, this parameter does not influence the position of the peaks, as expected, as the NW ensemble height remains constant. For t_off_ >800 nm, there is an effective chiral medium decrease; however, we believe that such NW pairs are rare in our samples and cannot significantly influence the results. In principle, the Au flux shadowing could be completely avoided by using nanolithography for defining the NW nucleation sites. Such pattern could be designed in such way that the shadow of one NW always falls in between the other NWs and never on them. Here, instead, our self-assembled technique has advances in terms of lower cost and scalability of the process (e.g. no need for large area electron beam lithography).


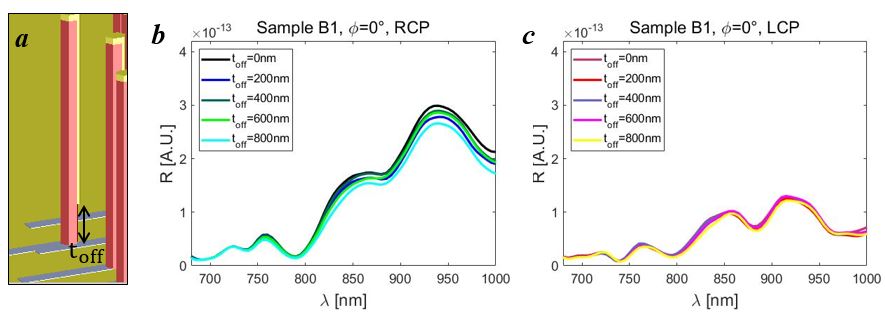


Figure S6: (a) Sketch of the shadowing problem in close NWs. Far-field reflection spectra for Sample B1, oriented at ϕ=0°, as a dependence on the Au sidewall offset t_off_: due to the shadowing effect: (a) at RCP excitation; (b) at LCP excitation.

Next, we investigate the influence of Au sidewall thickness on the chiral behavior. In Fig. S7 it is clear that the overall chiral behavior and position of the reflection peaks do not significantly differ for t_Au_>15nm. Below this limit, we believe that a too thin Au layer would decrease the field confinement effect in a sparse NW array, so that RCP excitation has a less pronounced interference feature, thus leading to lower CD. On the other side, the sidewall thickness increase leads to higher reflection of RCP in the highly reflective 950-1000 nm range, which can be further used for the optimization of CD_R_ in that range.


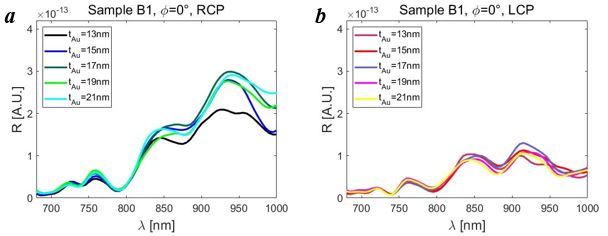


Figure S7: Far-field reflection spectra for Sample B1, oriented at ϕ=0°, as a dependence on Au sidewall thickness t_Au_: (a) at RCP excitation; (b) at LCP excitation.

In Fig. S8 we compare our previous results for Sample B1, with the ones where the NWs have three Au sidewalls. In the FDTD set-up, we keep all fabrication parameters of Sample B1, we rotate the NWs for 90°, and add three sidewalls of Au, where the middle one has thickness of 17 nm. We notice a similar chiral behavior, with RCP being more reflected than LCP, and having characteristic reflection peaks. The reflection intensity is somewhat lower compared to the experimentally investigated sample, which could be attributed to the increase of the effective NW diameter.


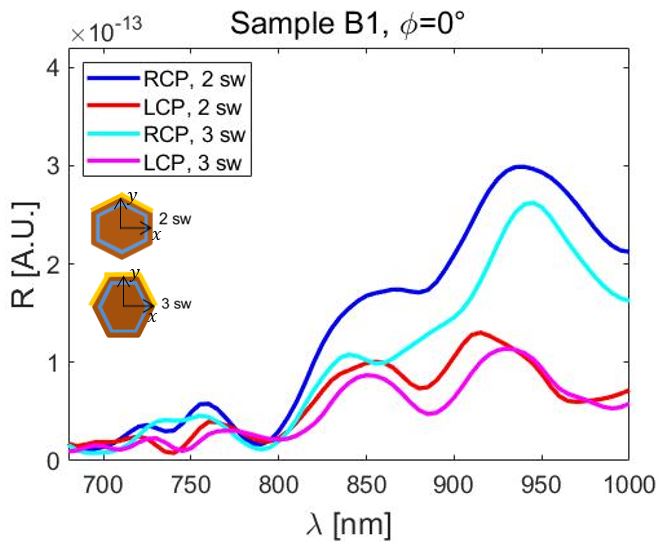


Figure S8: Far-field reflection spectra for Sample B1, oriented at ϕ=0°, for RCP and LCP excitations, when its two or three sidewalls (sw) are covered by 17 nm of Au.

Therefore, the advantages of our design lie in the fact that it exhibits chiral effects which are robust with respect to the fabrication variations, while the utilized lithography-free technique offers simpler and cost-effective development of chiral nanostructures.

Further, we focus on the near-field confinement across specific cross-sections for Sample B1, at ϕ=0°; we choose 940 nm as the excitation wavelength which leads to high CD_R_. We place two field profile monitors in xy plane across the whole FDTD domain; the first is placed at the middle of the NW height, while the second stands close to the NW bottom, i.e. 20 nm above the Au layer on the substrate. In Fig. S9 we show the electric field intensity enhancement (normalized to the one of the impinging electric field of 1 V/m). We see that in the middle of the NW ensemble, RCP light is more confined around the NWs, while for LCP excitation, there is a spread of the enhanced electric field between the neighboring NWs. As for the bottom monitor, there can be seen features of the NW shadow; the LCP excitation evidently leads to higher field intensity at this position than the RCP excitation.


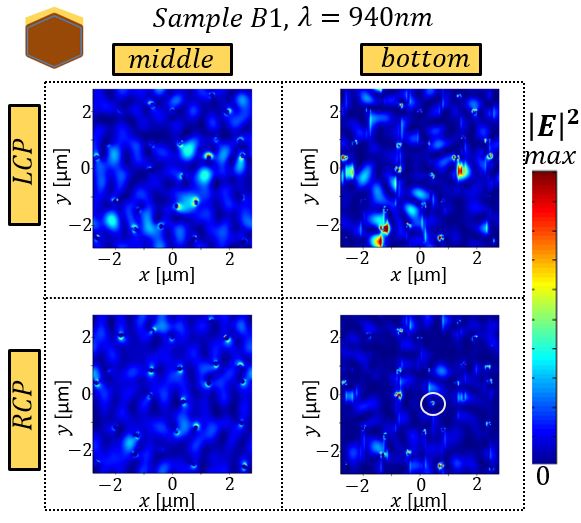


Figure S9: Field-profile monitors showing near-field intensity enhancement in xy-plane for Sample B1 at ϕ=0°; two monitors are placed in the middle of the NW length, and close to the bottom of the NW (20 nm above the Au layer), and LCP/RCP excitations are monitored at 940 nm.

Next, we place the xz- and yz- field profile monitors in the center of the NW circled in Fig. S9, and show the field confinement for LCP and RCP excitations, Fig. S10. In the xz-plane, RCP excitation produces a more regular interference pattern, which leads to the reflection maximum (Fig. 4a and Fig. 6b in the main manuscript); LCP excitation instead produces a complicated field pattern which becomes enhanced closer to the substrate. In order to visualize the influence of the Au layer, we plot the electric field confinement in yz-plane. LCP excitation confines the EM field to the dielectric side of the NW, transmitting it to the substrate, where it gets partially absorbed at this wavelength. RCP excitation, however, mostly couples to the Au shell, which is the reason behind its high specular reflection.


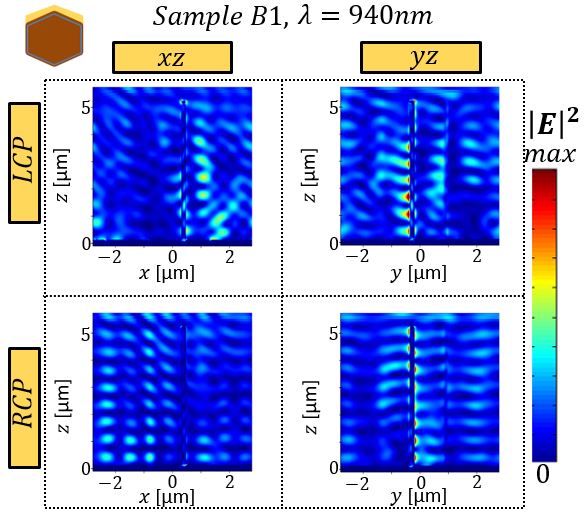


Figure S10: Field-profile monitors showing near-field intensity enhancement in xz- and yz-planes for Sample B1 at ϕ=0°; two monitors are placed in the middle of the NW shown in the circle of Fig. S5, and LCP/RCP excitations are monitored at 940 nm.

Furthermore, as the final extrinsic chirality figure of merit CD_R_ depends on the NW dimensions, we apply the model presented above to monitor the optimization of CD_R_ in the near-IR range. The fabrication technique implemented here and in our previous works provides ensembles with highly uniform dimensions, where the growth in the radial and vertical directions are linear functions of growth time [4]. We therefore take this linear function and investigate four more ensembles of NWs, keeping their aperiodic distribution (25 NWs), the surface density of Sample A1 (1 NW/µm^2^), and Au layer of 17 nm positioned at ϕ=0°. Samples NW1, NW2, NW3 and NW4 have the core diameter of 50 nm, 75 nm, 100 nm, and 125 nm, respectively, while their length is calculated from the linear dependence of [4], and varies from 1590 nm (NW1) to 4020 nm (NW4). In Fig. S11a, short and thin NWs lead to the highest specular reflection in the whole wavelength range, as expected due to the low absorption and high Au volume. However, their short length cannot introduce substantial difference in the light coupling with LCP or RCP excitation, thus leading to the lowest CD_R_, Fig. S11b. On the other side, the longest and thickest NW4 lead to the extremely low reflection in the range below 800 nm, due to the high absorption. However, in the range of high reflectivity, the oscillatory features lead to CD_R_ above 55%. In the intermediate wavelength range, the NW3 shows the highest CD_R_, due to the higher reflection with respect to the NW4, and longer effective medium length with respect to the NW1. We conclude that this model can be used to optimize the NW dimensions for the enhancement of CD_R_ in a given wavelength range.


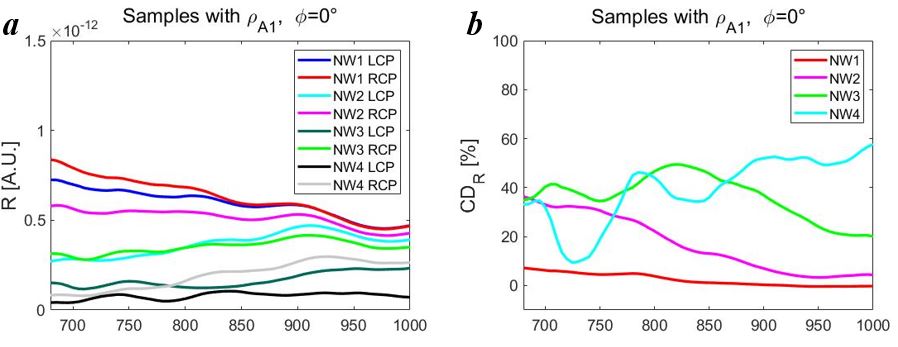


Figure S11: Investigation of NW1-4 samples with D=50-125 nm and L=1590-4020 nm, with surface density of 1NW/μ^2^, oriented at ϕ=0°: (a) far-field reflection spectra for LCP and RCP excitations; (b) CD_R_.

Owing to the BFAST approach, this model can be further used to extract and optimize absorption CD in a broadband wavelength range. In Fig. S12a-b we extract absorption of LCP and RCP for Sample A1 and different incidence angles. As expected, the absorption trend is opposite to the one of the reflection (Fig. 3, Fig. S5). We further calculate the absorption CD_A_ as the normalized absorption difference between LCP and RCP, as defined in [3] and measured by photo-acoustic technique. Here we underline that Sample A1 is similar to the Sample S1 from [3] in terms of the NW dimensions, while it has two instead of three Au sidewalls. In Fig. S12c, we notice that the overall dependence on the incidence angle θ agrees with the one reported at single wavelengths in [3]: across the almost whole wavelength range, CD_A_ reaches the maximum at θ=25° and decreases to lower values at θ=35° and finally at θ=45°. Moreover, tuning the incident angle and wavelength can lead to CD_A_ values more than twice as high as the ones reported in [3].


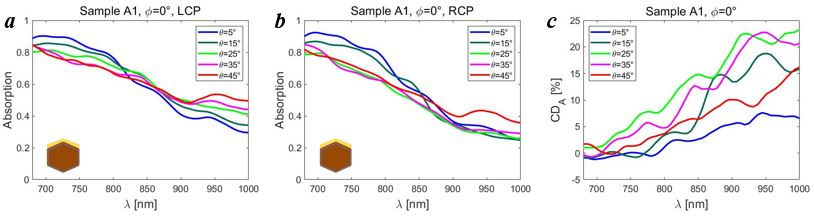


Figure S12: Absorption spectra of Sample A1 oriented at ϕ=0°, and excited by (a) LCP, and (b) RCP. (c) Broadband CD_A_ defined as normalized absorption difference between LCP and RCP [3].

**References**

[1] Lumerical Solutions, Inc. [http://www.lumerical.com/tcad-products/fdtd/](https://www.lumerical.com/tcad-products/fdtd/)

[2] Leahu, G.; Petronijevic, E.; Belardini, A.; Centini, M.; Li Voti, R.; Hakkarainen, T.; Koivusalo, E.; Guina, M.; Sibilia, C. Photo-acoustic spectroscopy revealing resonant absorption of self-assembled GaAs-based nanowires. Sci Rep. 2017, 7, 2833.

[3] Leahu, G.; Petronijevic, E.; Belardini, A.; Centini, M.; Sibilia, C.; Hakkarainen, T.; Koivusalo, E.; Rizzo Piton, M.; Suomalainen, S.; Guina, M. Evidence of Optical Circular Dichroism in GaAs-Based Nanowires Partially Covered with Gold. Adv. Opt. Mater. 2017, 5(16), 1601063.

[4] Koivusalo, E.; Hakkarainen, T.; Guina, M. Structural Investigation of Uniform Ensembles of Self-Catalyzed GaAs Nanowires Fabricated by a Lithography-Free Technique. Nanoscale 2017, Res. Lett. 12, 192.
